# Dual-Intended Deep Learning Model for Breast Cancer Diagnosis in Ultrasound Imaging

**DOI:** 10.3390/cancers14112663

**Published:** 2022-05-27

**Authors:** Nicolle Vigil, Madeline Barry, Arya Amini, Moulay Akhloufi, Xavier P. V. Maldague, Lan Ma, Lei Ren, Bardia Yousefi

**Affiliations:** 1 Fischell Department of Bioengineering, University of Maryland, College Park, MD 20742, USA; nvigil@umd.edu (N.V.); mbarry12@umd.edu (M.B.); lanma@umd.edu (L.M.); 2 Department of Radiation Oncology, City of Hope Comprehensive Cancer Center, Duarte, CA 91010, USA; aamini@coh.org; 3 Department of Computer Science, Perception Robotics and Intelligent Machines (PRIME) Research Group, University of Moncton, New Brunswick, NB E1A 3E9, Canada; moulay.akhloufi@umoncton.ca; 4 Department of Electrical and Computer Engineering, Laval University, Quebec City, QC G1V 0A6, Canada; xavier.maldague@gel.ulaval.ca; 5 Department of Radiation Oncology, University of Maryland School of Medicine, Baltimore, MD 21201, USA; lren@som.umaryland.edu

**Keywords:** ultrasound imaging, breast cancer, medical image analysis, dimensionality reduction, deep learning, radiomics

## Abstract

**Simple Summary:**

The findings of predictive and diagnostic systems in cancer are an intriguing topic for physicians and the oncologic community. Computer-aided decision (CAD) is vital for breast cancer diagnosis. It aids in higher accuracy and early, reliable diagnosis. To achieve such aims, diverse imaging modalities have been used and decision-making was facilitated by artificial intelligence and machine learning models. High-fidelity automated breast lesion finding, along with their corresponding radiomic feature biomarkers, can be delivered by a trained model. In this study, the potential impact of a machine learning model for detecting breast lesions and various radiomic biomarkers are examined. This study presents a model that automatically segments and extracts radiomics and can enable the clinical practice to find breast lesions while performing diagnosis concurrently.

**Abstract:**

Automated medical data analysis demonstrated a significant role in modern medicine, and cancer diagnosis/prognosis to achieve highly reliable and generalizable systems. In this study, an automated breast cancer screening method in ultrasound imaging is proposed. A convolutional deep autoencoder model is presented for simultaneous segmentation and radiomic extraction. The model segments the breast lesions while concurrently extracting radiomic features. With our deep model, we perform breast lesion segmentation, which is linked to low-dimensional deep-radiomic extraction (four features). Similarly, we used high dimensional conventional imaging throughputs and applied spectral embedding techniques to reduce its size from 354 to 12 radiomics. A total of 780 ultrasound images—437 benign, 210, malignant, and 133 normal—were used to train and validate the models in this study. To diagnose malignant lesions, we have performed training, hyperparameter tuning, cross-validation, and testing with a random forest model. This resulted in a binary classification accuracy of 78.5% (65.1–84.1%) for the maximal (full multivariate) cross-validated model for a combination of radiomic groups.

## 1. Introduction

Despite high survival rates and the current advancement of various imaging systems, used for diagnostic and treatment, breast cancer still accounts for the most fatal cancer among women, over 30% of the overall cancer death, according to the American Cancer Society and World Health Organization (WHO) reports [1,2]. X-ray mammography is the gold standard for breast cancer screening and is often used for the follow-up screening process as well. Other imaging modalities, such as magnetic resonance imaging, are more applicable to high-risk mutation cases and, due to being costly, are not considered for screening. Ultrasound (US) imaging is another common screening modality, which is highly dependent on the experience and expertise of its operator [3]. There are inherent limitations concerning medical imaging such as mammography and ultrasound due to being a projection imaging modality and a small field of view, which causes difficulties in finding microcalcification deep inside the breast lesions. This might cause a high recall rate for mammography, approximately 10%, or for digital breast tomosynthesis (DBT) [4,5]. Besides, the tissue superimposition increases false-positive rates in the diagnosis of benign solid mass, pseudo lesion, or calcifications as malignant tumors [6,7,8]. The prevalence of false-positive findings during breast imaging is known as the loudest criticism in the field [9,10]. In the USA, up to 20% of assessed masses were categorized as Breast Imaging Reporting and Data System (BI-RADS) category 3 (probably benign) and recommended for biopsy and short-interval follow up (6 months), while only 9–11% of biopsies prove to be malignant 36. On the other hand, without biopsy or frequent surveillance, diagnosis would be delayed and have an adverse effect on patients’ health. Consequently, it is eminently desired that next-generation breast imaging systems and screening practices must decrease unnecessary biopsies and false-positive call-backs, to reduce invasive procedures, radiation dose, cost, and avoidable anxiety in patients.

Computer-aided decision (CAD) systems showed undeniable help to physicians due to recent advancements in artificial intelligence (AI) technology [11,12,13,14]. Particularly, embedded advanced machine learning models, i.e., deep neural networks, helped boost the capabilities of CAD [15]. Important parameters for the diagnosis of breast cancer are related to tumor morphological information, which is often checked by physicians, and baseline characteristic features verified by CAD [13,14]. Imaging throughputs, or *radiomics*, decode information on the characteristics that were not visible to the naked/untrained eyes and can have significant effects on cancer diagnosis/prognosis [16,17,18,19,20,21,22,23].

Deep learning improves the capability of imaging throughputs for CADs, *deep-radiomics*, through transfer learning and extraction of hidden weights from the pre-trained models [24], or by creating new models to enhance the deep radiomics [25,26]. In both scenarios, high dimensional features aid CAD to enhance the ability to interpret the contents for non-imaging experts [27]. Similarly, segmentation of breast lesions in ultrasound images can be a challenging task that often involves physicians with trained eyes. CAD helped in the past to increase the accuracy of tumor findings through multiple methodologies, including deep learning with reasonably high accuracy [28,29]. Segmentation and classification of the tumors in medical imaging can be challenging due to multiple training processing and limited data in the medical field. Such problems, along with imbalanced training [26] or higher segmentation accuracy in the model [28], were addressed using different models’ configurations.

**Figure 1:**
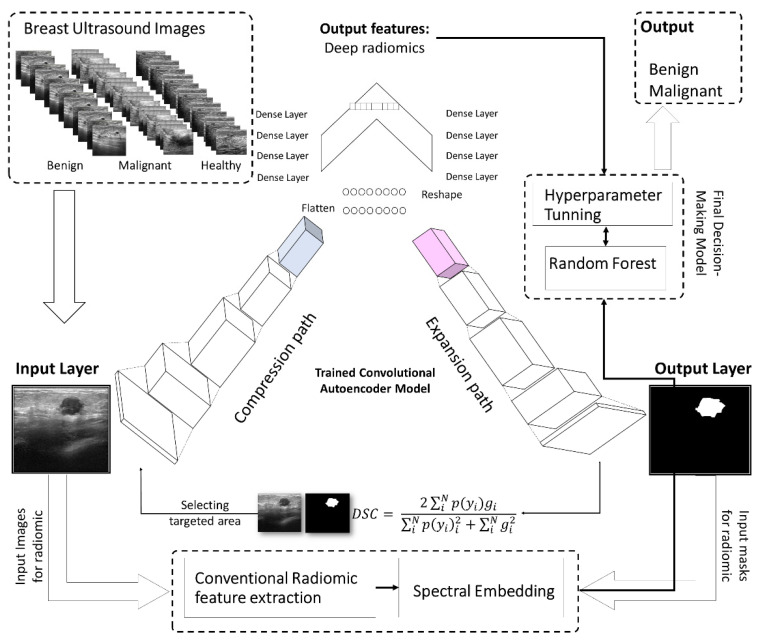
The workflow for automated breast lesion finding and breast cancer diagnostic system using deep autoencoder. The segmentation and radiomic extraction tasks are embedded in a single unit of the deep neural network, which mitigates the amount of data and training time required for the training of the model.

We tackle this challenge by designing a deep convolutional neural network model to segment and simultaneously extract deep-radiomics to be used for the classification of the breast lesion types (see Figure 1). The segmentation and radiomic extraction tasks are embedded in a single unit of the deep neural network, which mitigates the amount of data and training time required for the training of the model.

## 2. Materials and Methods

### 2.1. Study Data 

**Figure 2:**
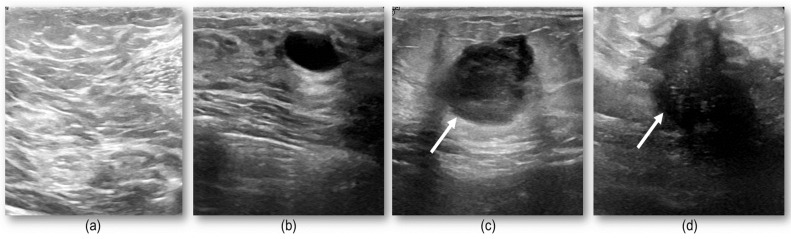
Four examples of ultrasound: (**a**) shows a normal case with no suspicious lesion, (**b**) depicts a benign nodule, while (**c**,**d**) show malignant tumors. These images show the challenge in discriminating different group of lesions with different textural complexities.

To test the feasibility of the proposed model, we conducted preliminary analyses examining an automated system for segmentation of the tumor in a large cohort of patients. The publicly available ultrasound images came from Baheya Hospital for Early Detection and Treatment of Women’s Cancer, Cairo, Egypt [30]. This imaging dataset consisted of 780 ultrasound images from 600 women ages 25 and 75 years old, collected in 2018. The collected ultrasounds represent different breast cancer states, normal, benign, and malignant. Figure 2 shows four examples of our ultrasound images for normal, benign, and malignant cases.

### 2.2. The Proposed Deep Learning Model

Radiomics have demonstrated a high potential to discover the characteristics of diseases in medical imaging that cannot be seen by the naked eye [18,19,20,21,22,23]. These imaging throughputs are typically a useful complement to clinical and biological covariates to enhance diagnostic capacity. Deep neural networks recently achieved certain improvements in diagnosis using conventional radiomics [31,32,33,34]. CAD systems often leverage receiving an ample number of covariates encapsulating the imaging attributes, while having too many radiomics inhibits the overall performance due to overfitting, known as the *curse of dimensionality* [34,35,36,37,38,39].

Hidden layers in the pre-trained models, such as ResNet [40], ImageNet [41], and VGG [42], are often used to extract deep features, which are high dimensional covariates and require the preprocess to prevent overfitting of our decision-making unit. Conventional feature selection or feature reduction methodology might not be the best solution to tackle this problem due to difficulties in interpreting closely bonded covariates in hidden weight which might be deduced as collinearity. A solution can be using a trained model to be susceptible to slowly varying features and succinctly reduce the dimensionality [36].

**Figure 3:**
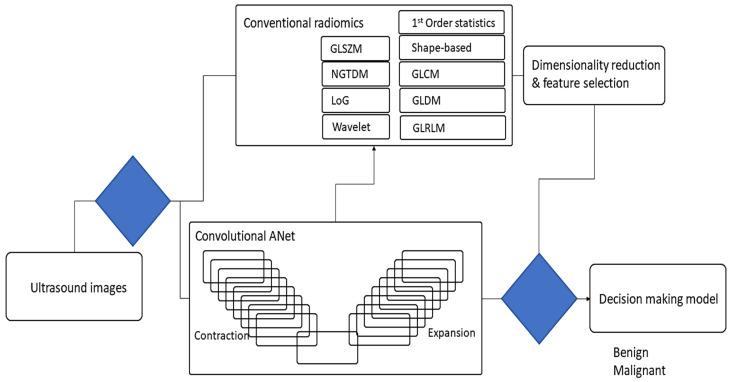
The scheme of the proposed multiple radiomic features generated from ultrasound images to segment and diagnose breast lesions.

In this study, we propose a convolutional deep autoencoder model with dual-purpose applications for segmenting and simultaneously extracting concise deep radiomics of the breast lesions. The generated radiomics are employed to diagnose breast cancer in ultrasound imaging. In the training phase, the segmented breast lesions have been provided to the model. After that, in the testing phase, the model performs segmentation while generating low dimension radiomics, known as latent space features. The loss function is measured as a distance between the model-segmented area and the ground truth. Our model’s parameters are optimized using a stochastic gradient descent algorithm trying to minimize the similarity distance and make sufficient segmentation. Figure 1 and Figure 3 show the workflow of the proposed approach in terms of model configuration and radiomic throughput, respectively.

The input to the model, $x\in {\mathbb{R}}^{n\;\times \;m}$, has the spatial dimensions of 512×512. Then this length decreases to η, which is the latent space dimension. $\eta =F_{e}\left(x\right)=a_{e}\left(Wx+b_{e}\right)$ is the abstracted representation for the input image, $a_{e}$ is the activation for compression path, and $b_{e}\;\mathrm{and}\;\;W\;$are the contracting path bias and weight matrices, respectively. $y=G_{d}\left(\eta \right)=a_{d}\left(W^{T}\eta \right)$ expands the latent representation of input to the original input spatial dimensions. y is the corresponding of x, and $a_{d}$ is the activation expansion path. A deep autoencoder is made of a multilayer model with some activations corresponding to each layer $a_{{\left(.\right)}_{i}}$, weights $W_{i}$, biases $b_{{\left(.\right)}_{i}},\;$and matrices minimizing $\left\{W_{i},b_{e_{i}}\right\}$. This is shown by $J_{\mathrm{AE}}=E_{x}\left[\ell \left(x,G_{d_{i}}\left(F_{e_{i}}\left(x\right)\right)\right)\right]$, where ℓ(.) is the model’s loss function, which is measured by the Dice similarity coefficient (DSC).

The proposed model decreases the dimensionality to 16, and after filtering the features, and weights, with lesser influence on our data, four deep-radiomics were extracted and used for training the random forest model. Discussion of computational complexity of the proposed model is in Appendix A.

This model is motivated by SPAER [43] configuration with slight modifications toward segmentation and alleviating sparsity in the latent representation by taking away the additional $\ell_{1}$ penalty term from the model. Figure 1 and Figure 3 display the configuration of the proposed deep learning model.

### 2.3. Conventional Radiomics in Breast US Imaging

Radiomic features can be divided into different categories. For example, first-order features, which include tissue density, shape features (i.e., volume and surface area), and texture features, describe spatial patterns of voxel intensities [5,7,9,11,12,13,14,15,16,17]. The proposed approach employs 354 radiomics features in nine categories: first-order statistics (FO), shape-based expression (SB), gray level co-occurrence matrix (GLCM), gray level dependence matrix (GLDM), gray level run length matrix (GLRLM), gray level size zone matrix (GLSZM), neighboring gray-tone difference matrix (NGTDM), Laplacian of Gaussian (LOG), and three-layer filtering wavelet features (see Table 1 and Supplementary Materials Table S1).

### 2.4. Dimensionality Reduction

The high dimensional radiomic data is addressed through two separated methodologies which interact by combining the outcome imaging biomarkers for the final decision-making unit, random forest. A deep neural network structure motivated by SPAER [43] proposed to reduce the dimensionality from 262,144 pixels to a 4 pixel-array biomarker. The second dimensionality reduction path involves conventional radiomics. A spectral embedding method and Laplacian eigenmaps [44], as a non-linear dimensionality reduction technique, mitigates the dimensionality from 354 radiomics to 12 radiomics.

### 2.5. Metrics for Breast Lesions Finding

Deep learning-based segmentation models have shown great performance in natural imaging [45]. In medicine, challenges are different from natural images and the models are often need multiple modifications in their network’s configurations. Among many structures, the UNet architecture has achieved exceptional capability in segmenting various targets or organs in many medical imaging modalities [46,47]. Reviewing the literature, we have chosen a slightly modified version of the original UNet [46] deep segmentation network to segment the ventricles. All models are trained from scratch using training and validation sets and evaluated on the testing set. The networks are trained with normalized input images (MRI slices) and their respective labels through their segmentation maps with stochastic gradient descent. 

Dice loss: The dice loss function, originally the Sørensen–Dice similarity coefficient in the 1940s, measures the similarity between two samples [48,49]. Dice score has been used for 3D segmentation of medical imaging [50]. The definition of DSC is presented as follows:

(1) $$\mathrm{DSC}=\frac{2\sum_{i}^{N}p\left(y_{i}\right)g_{i}}{\sum_{i}^{N}p{\left(y_{i}\right)}^{2}+\sum_{i}^{N}g_{i}^{2}}$$

where $p\left(y_{i}\right)$ is the probability of the outcome of the model for ith case, while the corresponding ground truth for that is $g_{i}$. This happens for N cases, the number of cases in the study. ${ℒ}_{\mathrm{Dice}}$ refers to the DSC obtained for the cases during one epoch. To train the model, we compute the Dice loss for every sample in our selected batch and then average over the batch.

### 2.6. Breast Lesions Detection

To detect the breast lesions, we used 550 cases from our overall cohort of patients and 100 cases for testing our model. We used this data pool concentrating on finding breast lesions and extracting deep-radiomics from the model simultaneously. For every group of patients, there was a random sampling which led to distributing the two categories of lesions, benign and malignant, equally across the input cases into the model.

### 2.7. Evaluation of Classifying Lesions

To evaluate the system’s performance in classifying lesions, we used metrics such as overall accuracy, precision, and recall through random forest classification. The number of trees in the forest, the maximum depth, and the random state in the tree were optimized by adjusting the hyperparameters for access to our data using the leave-one-out cross-validation technique. The selected group of abnormal cases, with breast lesions, are then analyzed by a classifier and evaluated by calculating and comparing their accuracy, precision, and recall. Finally, different tuning hyperparameters were chosen to optimize the model for diagnosis. The selected features from conventional radiomics and extracted deep-radiomics are concatenated with each other as input covariates into the model, while the ground truth of the classification is also given to the model to complete the benchmarking process. The model was trained with leave-one-out cross-validation and performed a t-test to check the statistical significance of the outcomes.

**Figure 4:**
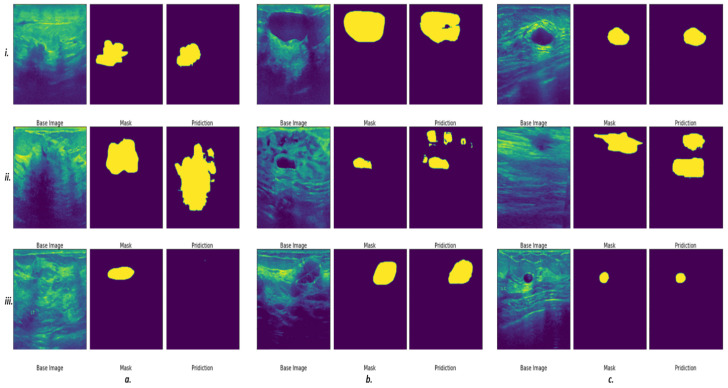
Preliminary results indicate a promising outcome of automated breast lesion segmentation. Some examples of successful (***a.i***,***c.i***,***b.iii***,***c.iii***), semi-successful (***b.i***,***a.ii***,***b.ii***,***c.ii***) and unsuccessful segmentations (***a.iii***) are presented.

To develop a deep learning-based automated image analysis pipeline, segmentation and extraction of high throughput quantitative measurements of breast cancer were performed in a hierarchical order. The current segmentation methodology of applying deep neural networks were improved to segment the breast lesions for ultrasound imaging and simultaneously extracted compressed deep radiomics. We developed a pipeline to incorporate a selection of image processing, deep learning, data analysis, and dimensionality reduction algorithms to expedite a series of steps to perform breast lesion segmentation, and deep/conventional radiomics (Figure 4). The pipeline includes training steps for segmenting breast lesions incorporating a deep autoencoder to train and perform segmentation. Following the model training, feature extraction was performed through conventional and our deep architecture imaging features to compute the breast lesions intensity and texture descriptors. In the testing phase, the pipeline underwent an evaluation during which parameters of deep autoencoder (i.e., batch size, learning rate, training epoch, etc.) were optimized in association with the outcome of interest. Deep-imaging biomarkers generated from our model will be compressed, while some approaches for selecting important features and dropping collinearity among the biomarkers need to be performed. Figure 3 demonstrates an overview of the workflow for automated diagnosis of breast cancer.

## 3. Results

The proposed CAD system involves a deep learning model with dual applications; we present the results based on the application of the model.

### 3.1. Segmentation of the Breast Lesions

**Figure 5:**
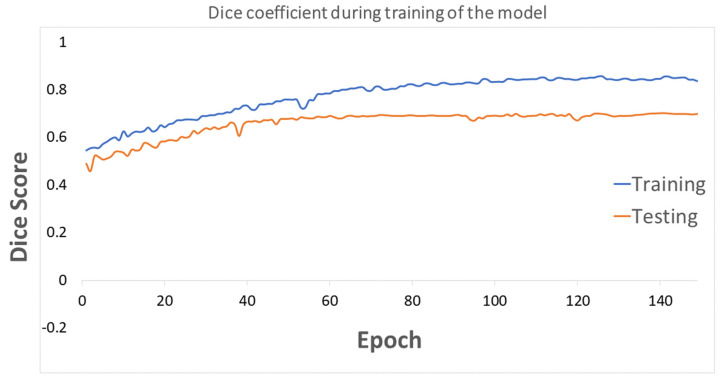
The Dice coefficient loss score is presented for training and testing the model during 150 epochs.

In total, 780 ultrasound images—437 benign, 210, malignant, and 133 normal—were used as the input for this system. This set was divided at random into training and testing sets, having 635 and 145 cases, respectively. We used the proposed deep learning structure for 2D ultrasound imaging segmentation. The input ultrasound images dimensions were 512 × 512, which were normalized before feeding them to the model. A convolutional layer, batch normalization, and a rectified activation linear unit (ReLu) layer were used for all input images. In the contracting path, all consecutive convolutional layers had a filter size of 3 × 3 and pooling layers of 2 × 2 shrunk the 512 × 512 input spatial dimension to 32 × 32 dimensions at the end of the encoder. Then, 16 filters were used in the convolutional layer to convolve the input ultrasound image, which used the same size padding with a 2 × 2 stride. The model applied a mirrored architecture for the decoder (expanding path) without skip connection (bridges) between two paths, as shown in Figure 1 and Figure 2. For upsampling data, a 3 × 3 kernel size deconvolved the contracted images. The networks overall number of trainable parameters was 190,279,473, with 512 maximum channels. The Adam optimizer was used for the training of all models with a modifying learning rate of $2\times 10^{-4}$ to$\;10^{-6}$. The models were trained for 150 epochs with a batch size of 8 for the training cohort of patients. The proposed deep learning model was implemented with the TensorFlow library in Python programming language [51,52]. The results of segmentation were attained throughout the inference process using Dice score similarity of the predicted lesions to the ground truth labels, which reached close to 85.7% and 70% for the training and validation sets, respectively. During training, the value of the Dice coefficient fluctuates approximately from 42% to 55% during the first 20 epochs, then increased and stabilized after the 70th epoch. To avoid overfitting, we kept training the proposed model in the 150 epochs range. Then, this trained model was used to segment the breast lesions for other patients’ strata and generated masks for them while extracting deep radiomics simultaneously. Figure 4 and Figure 5 show the results of segmentation with training and validation loss, and the accuracy of the training for 150 epochs, respectively. The result of computational time of the proposed model is presented in Table S1 in the Supplementary Material Section. In Figure 4, some examples of successful (***a.i****, **c.i**, **b.iii**, **c.iii***), partially-successful (***b.i, a.ii, b.ii, c.ii***) and unsuccessful segmentations (***a.iii***) are presented. The results indicate that the model tends to have more false positive errors than false negatives.

### 3.2. Conventional and Deep Latent Space Radiomics

In total, 354 conventional radiomics [20] were extracted via the original ultrasound images, and the proposed deep learning attained their matching masks for the validation sets. The images that went through the segmentation process were then used for extracting standard radiomics for their 2D targeted region of interest (ROI), i.e., solely for the breast lesion areas from our trained model. Out of all traditional radiomics, the dimensionality shrunk to 12 features using the spectral embedding approach. These twelve descriptors were selected based on the elbow method of choosing the best number of grouping features (Figure S1 in the Supplementary Material Section).

The proposed model consisted of five convolutional blocks and contained 190,279,473 trainable parameters, which preferred input images with 512 × 512 dimensions. After that, the input image goes through 32 filters with 3 × 3 kernels. The re-scaling process of the dimensions through the model was from 512, 256, 128, 64, 32, 16, and 8, while the dimensions grew from 8, 16, 32, 64, 128, 256, and 512. A hierarchy of dense layers flattened and compressed the data in the middle of the model from 262,144 to 16, which produced 16 deep latent space features. Out of 16, four features were selected, and the rest were discarded due to having minimum variations or being zero, which were used for classification.

Table 2 reports the classification power capability of a multivariate classifier, random forest, to categorize breast lesions into benign and malignant classes. This leads to an automatic diagnostic outcome for the model. The accuracies of the models are measured with the respect to input imaging throughputs, and conventional or deep radiomics. Statistical distribution of each class of features was measured using the Wilcoxon test and regression analysis, which determined a significant statistical strength for discriminating benign versus malignant with a *p*-value < 0.005 (Figures S2–S5 in the Supplementary Material Section).

### 3.3. Optimizing the Classifier’s Hyperparameters

**Figure 6:**
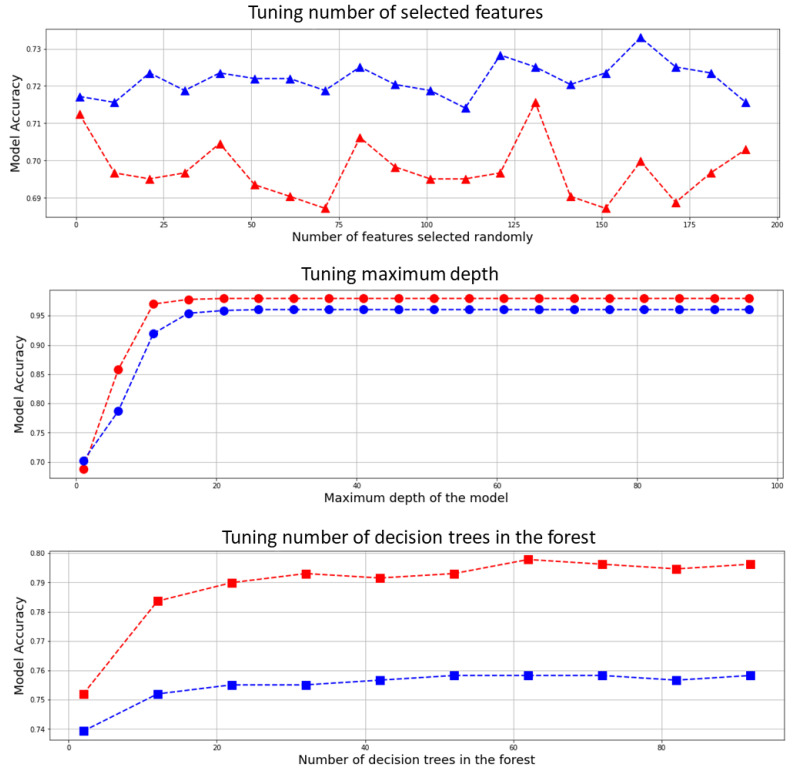
Hyperparameter tuning for the random forest, blue curves are representing deep radiomics and red curves show conventional radiomics fed to the model for the tuning using leave-one-out cross validation.

Hyperparameters of the random forest model were playing important role in classifying breast lesions. These hyperparameters consisted of the number of decision trees in the forest, the maximum depth of the model, and the number of features considered by each tree when splitting a node. To obtain the optimum hyperparameters, we tuned the model for all the possible degrees of freedom through an empirical evaluation for each combination. Figure 6 presents the variations in accuracy corresponding to each change in hyperparameter.

To tune the hyperparameters, many iterations of the entire leave-one-out cross-validation process are performed, each time using different model settings for the three aforementioned hyperparameters, i.e., number of the trees, max number of levels in each decision tree (maximum depth), and random state. All the models were then compared to select the best and train it on the training set, while one case was temporarily eliminated. Moreover, a grid search algorithm was employed through K-fold (k = 5) cross-validation to find the optimum hyperparameters for this model, which confirm the selected hyperparameters (Section S1 and Table S3 in the Supplementary Material Section).

### 3.4. Classification Performance of the Proposed Model

To measure the accuracy of the model quantitatively, we utilized the clinical diagnosis as the gold standard or ground truth for our classification. The ground truth images were labeled as 0 for benign and 1 for malignant lesions. The classification was then performed based on binary classification for two groups of labels. There is a third category of the participants that have no lesions detected for them and are healthy cases.

**Figure 7:**
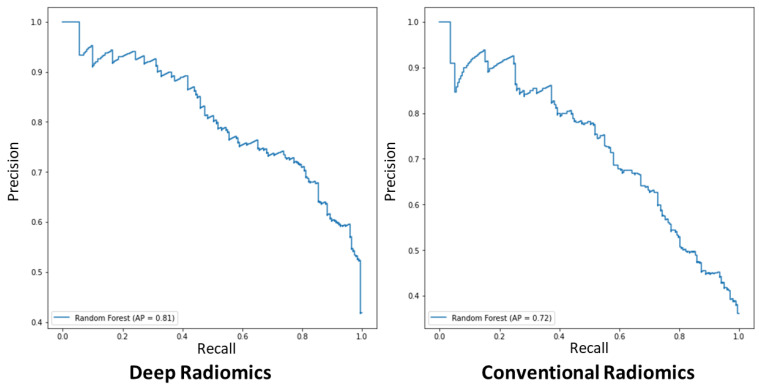
The representation of conventional and deep radiomic features for the training random forest model for diagnosis of breast cancer with the respect to precision and recall.

We created two groupings of patients based on imaging throughputs and classified them by a random forest classifier. Three imaging biomarkers were incorporated to classify the lesions as benign and malignant, i.e., conventional radiomics, deep radiomics, and a combination of both groups. We classified other input cases based on four proposed deep learning deep latent space radiomic descriptors, twelve conventional radiomics, and lastly, a combination of the two and compared them with the ground truth data from clinical assessment. To examine the appropriateness of imaging biomarkers for diagnosing breast cancer patients, we performed a classification with an optimized multivariate random forest model with leave-one-out cross-validation. Table 2 and Figure 7 show the cross-validated accuracy of the proposed method with the respect to hyperparameters’ adjustment, respectively.

The proposed model could reach a higher accuracy by adjusting the hyperparameters. In Table 2, the accuracy of the model reached to 85.3% (65.3–89.1%) for No. est. = 22, Max depth = 6, and Rand. state = 90. However, we reported the accuracy of the classifier with the respect to hyperparameters in order to avoid overfitting. The selected multivariate binary-class classification model for benign versus malignant detected breast cancer lesions was the conventional radiomics with No. est. = 22, Max depth = 5, and Rand. state = 80, which resulted in an accuracy of 78.8% (64.7–85.5%), and combined radiomics for the combination of both radiomic types (conventional radiomics and deep radiomics) resulted in 78.5% (64.5–83.9%) and 78.5% (65.1–84.1%), respectively.

Conventional radiomics with hyperparameters of No. est. = 10, Max depth = 2, and Rand. state = 10 for the random forest model showed the lowest binary-class classification accuracy of 69.03% (52.9–74.3%), and deep and combined models to detect malignant lesions, which yielded 67.7% (45.5–72.7%) and 71.7% (52.9–74.3%), respectively. This might be due to a low number of random observations and feature reduction in the model, which tends to weaken the classifier to generalize the model during the training stage. This trend is also observed, yet improved in the models trained with No. est. = 25, Max depth = 3, and Rand. state = 30 parameters for the classifier with 73.1% (59.8–78.8%), 73.3% (59.9–76.9%), and 73.2% (59.8–76.7%) accuracies for conventional, deep, and combined radiomics, respectively. The model’s hyperparameters, No. est. = 15, Max depth = 4, and Rand. state = 65, showed the middle-high range accuracy, 75.6% (62.4–82.05%), 75.2% (62.4–81.2%), and 75.2% (62.4–80.4%) for three aforementioned types of imaging throughputs, respectively. We also assessed the statistical distinction of the maximal accuracy, Conv. Radiomics No. est. = 22, Max depth = 5, and Rand. state = 80, with other hyperparameters’ variations applying a two-tailed t-test (see Table 2). The maximal model showed statistical significance with the entire model, e.g., the maximal model versus the combined model yield difference of t-statistic = 29.8 (*p*-value < 0.0005). Moreover, we calculated Kappa coefficients for validating our inter-rater reliability of the classifications for different tuning hyperparameters of the random forest model for conventional and deep radiomics, which yielded to 74.0% (± 12.0), corresponding to average and standard deviation of the Kappa coefficient for the reported accuracy of the model (See Table 2). Figure 7 shows the precision–recall curve for each class for conventional and radiomic features separately. For binary-class classification using deep-radiomics, the graph showed higher average precision (AP), reaching 81%, while conventional radiomics yield 72% AP. This indicates that with deep radiomics, the maximal model can classify the benign versus malignant with more precision.

## 4. Discussion

This study proposed an automatic approach to detect and diagnose breast lesions concurrently employing a self-designed deep neural network model [53]. The model generates deep radiomics and segments of breast cancer lesions, which helped to extract standard radiomics in ultrasound images. The model is self-sufficient and creates low-dimensional deep radiomics and integrates them with selected conventional radiomics for diagnostic purposes. This study demonstrated the possibility of a diagnosis of breast cancer lesions using a fast, non-invasive, and cost-effective ultrasound imaging-based diagnostic system as a recommender system to be used as a tool for physicians and radiologists to increase the precision.

The application of the proposed model implied the viability of a dual intended automated system with segmentation and extraction of low-dimensional deep radiomics from ROI. The proposed model exhibited relatively considerable accuracy compared to the state-of-art models, i.e., ASS-GANs [54], and W-Net [55], yet the combination of concurrent low-dimensional radiomics increases the contribution of this model, which significantly reduces the training process and required data compared cascading multiple models. Similarly, our model exhibited considerable growth in model performance for classifying breast cancer patients from benign cases (Figure 7, and Table 2). The proposed model in this study follows the previously mentioned methodologies [43], to design models for generating deep radiomics with low dimensionality, thereby surpassing the possibility of overfitting in our model and the issue of the *curse of dimensionality*. The proposed model gave low-dimensional deep radiomics through its latent space projection, while instantaneously segmenting breast lesions in ultrasound images.

The proposed model exhibits some limitations, one limitation is due to the absence of clinical information for this study, the clinical information and demographics were not included in the diagnostic decisions. In addition, most women undergo screening mammograms, and ultrasound is often utilized for confirmation of an abnormality on ultrasound or to evaluate symptoms. This proposed system is a CAD model to assist this procedure for ultrasound, and for the mammography there should be a comparable model to perform CAD, i.e., [56,57].

Despite a considerable number of cases and features to project the attributes for this study, there is a necessity for incorporating clinical input to achieve high diagnostic reliability in this system. Moreover, to achieve high validity and generalizability of the system, an independent imaging set seems to be required. In addition, exploring other deep learning configurations may lead to high discriminative capability in ultrasound images. Furthermore, there is a possibility to use an end-to-end deep neural mechanism to reduce additional decision-making models. The application of the sophisticated manifold learning and dimensionality reduction models is seemingly appealing as an alternative to the current conventional radiomics. This model designed and trained for quality ultrasound images, and may exhibit failing of segmenting lesions in poor sonographic images. This can be an intriguing topic for future investigation. The proposed model, like other CADs, acts as a recommendation system to physicians and is dependent on the training. To provide higher reliability, generalizability, and accuracy for more difficult cases, i.e., fibroepithelial lesion, or intraductal papilloma, the system must be trained with a sufficient number of the relevant ultrasound images.

The proposed model offers several advantages. First, using the proposed deep learning model to extract deep radiomics and concurrently segmenting the breast lesions delivers an effective feature extraction technique with much lesser data and training duration. To the best of our knowledge, this is the first time such a model has been used in breast cancer diagnosis in ultrasound imaging. In addition, this prevents pre-trained models with higher dimensional feature extraction and consequent overfitting of the decision-making model. Second, the dual application of the proposed deep learning model alleviates the complexity of the model to use separate models for segmentation and feature extraction. Third, our proposed model impedes applying human-engineered feature selection/reduction.

## 5. Conclusions

This study presented two methods to tackle the problem of the *curse of dimensionality* involving conventional and deep convolutional neural network-driven imaging biomarkers, radiomics. Moreover, the proposed deep learning model has exhibited a dual-purpose behavior, which automatically segmented ultrasound lesions and extracted low-dimensional radiomics, four deep-radiomics. The model was trained to segment the breast lesions while extracting radiomic features simultaneously. For traditional radiomics, 354 features were extracted from automated segmented breast lesions using a radiomic library, and its dimensionality decreased more than 29 times to 12 imaging biomarkers by applying the spectral mapping algorithm. To make the final diagnosis decision, a random forest model has been used that trained, tuned, and tested to classify segmented lesions to malignant and benign categories. The proposed system has been trained and validated using 780 ultrasound images to segment and diagnose the segmented breast lesions. The accuracy of the random forest model obtained through leave-one-out cross-validation yielded 78.5% (65.1–84.1%) for the maximal (full multivariate) cross-validated model, while the model for conventional- and deep-radiomics gave the accuracies of 78.8% (64.7–85.5%) and 78.5% (64.5–83.9%), respectively. In future work, we would expand our analysis for an independent dataset by combining it with other types of data to tackle the system’s generalizability and reliability.

## Figures and Tables

**Table 1:** The proposed application of conventional and modified deep learning texture descriptors.

Type of Texture Analysis	Categories
Conventional Radiomics	First-Order Statistics (FO)
	Shape-Based Expression (SB)
	Gray Level Co-Occurrence Matrix (GLCM)
	Gray Level Dependence Matrix (GLDM)
	Gray Level Run Length Matrix (GLRLM)
	Gray Level Size Zone Matrix (GLSZM)
	Neighboring Gray Tone Difference Matrix (NGTDM)
	Laplacian Of Gaussian (LOG)
	Wavelet
Deep Learning model-made Radiomics	Deep Convolutional Autoencoders

**Table 2:** Results of automated diagnosis of breast cancer in ultrasound imaging based on the classification of benign versus malignant patients with leave-one-out cross-validation. Convolutional and Deep learning radiomics are presented by conv and deep, respectively. Also, number of the trees (No. est.), maximum depth (Max. depth), and random state (Rand. state).

Accuracy of Different Multivariate Models for Breast Cancer Diagnosis in Ultrasound Images					
Methods	Hyperparameter	Radiomics	Classification Accuracy ^1^ (%)	Kappa Coefficient (κ)	*t*-Test ^2^ *t*-Statistic, Two-Tailed *p*-Value
Random Forest	No. est. = 10	Conv	69.03 (52.9–74.3)	57.4 (±18.5)	–
	Max depth = 2	Deep	67.7 (45.5–72.7)	59.8 (±17.2)	10.2, <0.0005
	Rand. state = 10	Conv + Deep	71.7 (52.9–74.3)	60.1 (±17.1)	6.01, <0.0005
	No. est. = 25	Conv	73.1 (59.8– 78.8)	65.1 (±14.6)	–
	Max depth = 3	Deep	73.3 (59.9–76.9)	64.7 (±14.7)	10.1, <0.0005
	Rand. state = 30	Conv + Deep	73.2 (59.8–76.7)	64.9 (±14.8)	7.9, <0.0005
	No. est. = 15	Conv	75.6 (62.4–82.05)	69.1 (±13.6)	–
	Max depth = 4	Deep	75.2 (62.4–81.2)	69.8 (±13.5)	16.9, <0.0005
	Rand. state = 65	Conv + Deep	75.2 (62.4–80.4)	69.7 (±13.2)	19.7, <0.0005
	No. est. = 22	Conv	78.8 (64.7–85.5)	73.7 (±12.5)	–
	Max depth = 5	Deep	78.5 (64.5–83.9)	73.9 (±12.2)	21.6, <0.0005
	Rand. state = 80	Conv + Deep	78.5 (65.1–84.1)	74.0 (±12.0)	29.8, <0.0005
	No. est. = 22	Conv	83.9 (67.9–90.2)	79.2 (±12.8)	–
	Max depth = 6	Deep	84.9 (66.6–90.9)	78.4 (±12.1)	30.7, <0.0005
	Rand. state = 80	Conv + Deep	84.6 (66.8–89.9)	79.6 (±12.2)	37.8, <0.0005
	No. est. = 22	Conv	85.1 (65.9–89.9)	77.9 (±13.3)	–
	Max depth = 6	Deep	83.7 (65.1–89.8)	78.1 (±12.5)	33.2, <0.0005
	Rand. state = 90	Conv + Deep	85.3 (65.3–89.1)	78.8 (±12.7)	39.1, <0.0005

^1^ Classification accuracy reported by median (± IQR) (interquartile range—IQR). ^2^ *t*-test calculated for each method versus maximal accuracy.

## Data Availability

In this study, we used publicly available datasets [30].

## Supplementary Materials

The following are available online at https://www.mdpi.com/article/10.3390/cancers14112663/s1. Table S1. Computational time of the designed deep learning model. Table S2. More detailed information about the Radiomic features. Table S3. The hyperparameter scoring obtained by grid search and 5-fold cross validation of the model and their scores and optimization time. Figure S1. Elbow technique to calculate the distortion score and find out the optimum number of the cluster for conventional radiomics. Figure S2. Regression analysis for benign and malignant lesions for four conventional radiomics and four deep radiomics. Figure S3. Distribution of conventional radiomics and their effect on diagnosis for malignant and benign lesions. Figure S4. The distribution of Deep radiomics and their classification strength. Figure S5. The receiver operating characteristic (ROC) curves for different multivariant models using conventional and deep radiomic features.

## Appendix A. Computational Complexity of the Proposed Model

The major computational complexity of the convolutional neural networks concerns convolutional layers, which leads to 90–95% of the overall complexity of the model [58,59]. The proposed convolutional autoencoder also follows the same complex tendency due to the designed convolutional layers. The given complexity of the proposed structure can be approximated as:

(A1) $$O\left(\overset{L}{\underset{i=1}{\sum }}c_{i-1}.s_{i}^{2}.\;c_{i}.m_{i}^{2}\right)$$

where i is the convolutional layer index and L is the overall number of convolutional layers, which is 5 for the presented model. $c_{i}$ presents the channel number of the *i*th layer index and spatial kernel size is shown by $s_{i}$ (all consecutive convolutional layers had a filter size of 3 × 3), while $m_{i}$ denotes the spatial size of the output feature maps. In our model, 16 filters were used in the convolutional layer to convolve the input ultrasound image, which used the same size padding with a 2 × 2 stride. Our model followed the standard network and have $m_{i}$ of each layer, as half-resolution of the input image, which is also halved.

## References

[1] Siegel R.L., Miller K.D., Fuchs H.E., Jemal A. (2021). Cancer statistics. A Cancer J. Clin..

[2] World Health Organisation (2018). Cancer—Key Facts. http://www.who.int/news-room/fact-sheets/detail/cancer.

[3] Jalalian A., Mashohor S., Mahmud R., Karasfi B., Saripan M.I., Ramli A.R.B. (2017). Foundation and methodologies in computer-aided diagnosis systems for breast cancer detection. EXCLI J..

[4] Scott A.M., Lashley M.G., Drury N.B., Dale P.S. (2019). Comparison of Call-Back Rates between Digital Mammography and Digital Breast Tomosynthesis. Am. Surg..

[5] Pisano E.D., Gatsonis C., Hendrick E., Yaffe M., Baum J.K., Acharyya S., Conant E.F., Fajardo L.L., Bassett L., D’Orsi C. (2005). Diagnostic Performance of Digital versus Film Mammography for Breast-Cancer Screening. N. Engl. J. Med..

[6] Mario J., Venkataraman S., Dialani V., Slanetz P.J. (2015). Benign breast lesions that mimic cancer: Determining radiologic-pathologic concordance. Appl. Radiol..

[7] Morrell S., Barratt A., Irwig L., Howard K., Biesheuvel C., Armstrong B. (2009). Estimates of overdiagnosis of invasive breast cancer associated with screening mammography. Cancer Causes Control.

[8] Puliti D., Paci E. (2009). The other side of technology: Risk of overdiagnosis of breast cancer with mammography screening. Futur. Oncol..

[9] Alagoz O., Chhatwal J., Burnside E.S. (2013). Optimal Policies for Reducing Unnecessary Follow-up Mammography Exams in Breast Cancer Diagnosis. Decis. Anal..

[10] Berg W.A. (2020). Reducing Unnecessary Biopsy and Follow-up of Benign Cystic Breast Lesions. Radiology.

[11] Cho K.R., Seo B.K., Woo O.H., Song S.E., Choi J., Whang S.Y., Park E.K., Park A.Y., Shin H., Chung H.H. (2016). Breast Cancer Detection in a Screening Population: Comparison of Digital Mammography, Computer-Aided Detection Applied to Digital Mammography and Breast Ultrasound. J. Breast Cancer.

[12] Theek B., Magnuska Z., Gremse F., Hahn H., Schulz V., Kiessling F. (2020). Automation of data analysis in molecular cancer imaging and its potential impact on future clinical practice. Methods.

[13] Le E.P.V., Wang Y., Huang Y., Hickman S., Gilbert F.J. (2019). Artificial intelligence in breast imaging. Clin. Radiol..

[14] Chabi M.-L., Borget I., Ardiles R., Aboud G., Boussouar S., Vilar V., Dromain C., Balleyguier C. (2012). Evaluation of the Accuracy of a Computer-aided Diagnosis (CAD) System in Breast Ultrasound according to the Radiologist’s Experience. Acad. Radiol..

[15] Burt J.R., Torosdagli N., Khosravan N., Raviprakash H., Mortazi A., Tissavirasingham F., Hussein S., Bagci U. (2018). Deep learning beyond cats and dogs: Recent advances in diagnosing breast cancer with deep neural networks. Br. J. Radiol..

[16] Shen W.-C., Chang R.-F., Moon W.K., Chou Y.-H., Huang C.-S. (2007). Breast Ultrasound Computer-Aided Diagnosis Using BI-RADS Features. Acad. Radiol..

[17] Kim S.-Y., Choi Y., Kim E.-K., Han B.-K., Yoon J.H., Choi J.S., Chang J.M. (2021). Deep learning-based computer-aided diagnosis in screening breast ultrasound to reduce false-positive diagnoses. Sci. Rep..

[18] Gillies R.J., Kinahan P.E., Hricak H. (2016). Radiomics: Images Are More than Pictures, They Are Data. Radiology.

[19] Lambin P., Rios-Velazquez E., Leijenaar R., Carvalho S., van Stiphout R.G.P.M., Granton P., Zegers C.M.L., Gillies R., Boellard R., Dekker A. (2012). Radiomics: Extracting more information from medical images using advanced feature analysis. Eur. J. Cancer.

[20] Aerts H., Velazquez E.R., Leijenaar R.T.H., Parmar C., Grossmann P., Carvalho S., Bussink J., Monshouwer R., Haibe-Kains B., Rietveld D. (2014). Data from: Decoding tumour phenotype by noninvasive imaging using a quantitative radiomics approach. Nat. Commun..

[21] Lambin P., Leijenaar R.T.H., Deist T.M., Peerlings J., de Jong E.E.C., van Timmeren J., Sanduleanu S., Larue R.T.H.M., Even A.J.G., Jochems A. (2017). Radiomics: The bridge between medical imaging and personalized medicine. Nat. Rev. Clin. Oncol..

[22] Wei M., Du Y., Wu X., Su Q., Zhu J., Zheng L., Lv G., Zhuang J. (2020). A Benign and Malignant Breast Tumor Classification Method via Efficiently Combining Texture and Morphological Features on Ultrasound Images. Comput. Math. Methods Med..

[23] Yousefi B., Sharifipour H.M., Maldague X.P.V. (2021). A Diagnostic Biomarker for Breast Cancer Screening via Hilbert Embedded Deep Low-Rank Matrix Approximation. IEEE Trans. Instrum. Meas..

[24] Sun Q., Lin X., Zhao Y., Li L., Yan K., Liang D., Sun D., Li Z.-C. (2020). Deep Learning vs. Radiomics for Predicting Axillary Lymph Node Metastasis of Breast Cancer Using Ultrasound Images: Don’t Forget the Peritumoral Region. Front. Oncol..

[25] Pang T., Wong J.H.D., Ng W.L., Chan C.S. (2020). Deep learning radiomics in breast cancer with different modalities: Overview and future. Expert Syst. Appl..

[26] Fei X., Zhou S., Han X., Wang J., Ying S., Chang C., Zhou W., Shi J. (2021). Doubly supervised parameter transfer classifier for diagnosis of breast cancer with imbalanced ultrasound imaging modalities. Pattern Recognit..

[27] Yap M.H., Edirisinghe E., Bez H. (2010). Processed images in human perception: A case study in ultrasound breast imaging. Eur. J. Radiol..

[28] Pan P., Chen H., Li Y., Cai N., Cheng L., Wang S. (2020). Tumor segmentation in automated whole breast ultrasound using bidirectional LSTM neural network and attention mechanism. Ultrasonics.

[29] Zhou Y., Chen H., Li Y., Liu Q., Xu X., Wang S., Yap P.-T., Shen D. (2020). Multi-task learning for segmentation and classification of tumors in 3D automated breast ultrasound images. Med. Image Anal..

[30] Al-Dhabyani W., Gomaa M., Khaled H., Fahmy A. (2019). Dataset of breast ultrasound images. Data Brief.

[31] Suk H.I., Shen D. Deep learning-based feature representation for AD/MCI classification. Proceedings of the International Conference on Medical Image Computing and Computer-Assisted Intervention.

[32] Virmani J., Agarwal R. (2020). Deep feature extraction and classification of breast ultrasound images. Multimed. Tools Appl..

[33] Antropova N., Huynh B.Q., Giger M.L. (2017). A deep feature fusion methodology for breast cancer diagnosis demonstrated on three imaging modality datasets. Med. Phys..

[34] Yousefi B., Kawakita S., Amini A., Akbari H., Advani S., Akhloufi M., Maldague X., Ahadian S. (2021). Impartially Validated Multiple Deep-Chain Models to Detect COVID-19 in Chest X-ray Using Latent Space Radiomics. J. Clin. Med..

[35] Yousefi B., LaRiviere M.J., Cohen E.A., Buckingham T.H., Yee S.S., Black T.A., Chien A.L., Noël P., Hwang W.-T., Katz S.I. (2021). Combining radiomic phenotypes of non-small cell lung cancer with liquid biopsy data may improve prediction of response to EGFR inhibitors. Sci. Rep..

[36] Yousefi B., Akbari H., Maldague X. (2020). Detecting Vasodilation as Potential Diagnostic Biomarker in Breast Cancer Using Deep Learning-Driven Thermomics. Biosensors.

[37] Yousefi B., Jahani N., Lariviere M.J., Cohen E., Hsieh M.-K., Luna J., Chitalia R.D., Thompson J.C., Carpenter E.L., Katz S.I. (2019). Correlative hierarchical clustering-based low-rank dimensionality reduction of radiomics-driven phenotype in non-small cell lung cancer. Medical Imaging 2019: Imaging Informatics for Healthcare, Research, and Applications.

[38] Ha S., Choi H., Paeng J.C., Cheon G.J. (2019). Radiomics in Oncological PET/CT: A Methodological Overview. Nucl. Med. Mol. Imaging.

[39] Bouveyron C. (2020). High-Dimensional Statistical Learning and Its Application to Oncological Diagnosis by Radiomics. Healthcare and Artificial Intelligence.

[40] He K., Zhang X., Ren S., Sun J. Deep residual learning for image recognition. Proceedings of the IEEE Conference on Computer Vision and Pattern Recognition.

[41] Deng J., Dong W., Socher R., Li L.J., Li K., Fei-Fei L. Imagenet: A large-scale hierarchical image database. Proceedings of the 2009 IEEE Conference on Computer Vision and Pattern Recognition.

[42] Simonyan K., Zisserman A. (2014). Very deep convolutional networks for large-scale image recognition. arXiv.

[43] Yousefi B., Akbari H., Hershman M., Kawakita S., Fernandes H., Ibarra-Castanedo C., Ahadian S., Maldague X. (2021). SPAER: Sparse Deep Convolutional Autoencoder Model to Extract Low Dimensional Imaging Biomarkers for Early Detection of Breast Cancer Using Dynamic Thermography. Appl. Sci..

[44] Belkin M., Niyogi P. (2003). Laplacian Eigenmaps for Dimensionality Reduction and Data Representation. Neural Comput..

[45] Fu Y., Lei Y., Wang T., Curran W.J., Liu T., Yang X. (2021). A review of deep learning based methods for medical image multi-organ segmentation. Phys. Med..

[46] Ronneberger O., Fischer P., Brox T. U-net: Convolutional networks for biomedical image segmentation. Proceedings of the International Conference on Medical Image Computing and Computer-Assisted Intervention.

[47] Zhou Z., Rahman Siddiquee M.M., Tajbakhsh N., Liang J. (2018). Unet++: A nested u-net architecture for medical image segmentation. Deep Learning in Medical Image Analysis and Multimodal Learning for Clinical Decision Support.

[48] Sorensen T. (1948). A method of establishing groups of equal amplitude in plant sociology based on similarity of species and its application to analyses of the vegetation on Danish commons. Biol. Skar..

[49] Dice L.R. (1945). Measures of the Amount of Ecologic Association Between Species. Ecology.

[50] Milletari F., Navab N., Ahmadi S.-A. V-net: Fully convolutional neural networks for volumetric medical image segmentation. Proceedings of the 2016 Fourth International Conference on 3D Vision (3DV).

[51] Google (2020). Python 3 Google Compute Engine Backend, T4, and P100 GPU and 27.4 Gb RAM.

[52] Abadi M., Barham P., Chen J., Chen Z., Davis A., Dean J., Zheng X. Tensorflow: A system for large-scale machine learning. Proceedings of the 12th Symposium on Operating Systems Design and Implementation.

[53] Khan A.I., Shah J.L., Bhat M.M. (2020). CoroNet: A deep neural network for detection and diagnosis of COVID-19 from chest x-ray images. Comput. Methods Programs Biomed..

[54] Zhai D., Hu B., Gong X., Zou H., Luo J. (2022). ASS-GAN: Asymmetric semi-supervised GAN for breast ultrasound image segmentation. Neurocomputing.

[55] Gare G.R., Li J., Joshi R., Magar R., Vaze M.P., Yousefpour M., Rodriguez R.L., Galeotti J.M. (2021). W-Net: Dense and diagnostic semantic segmentation of subcutaneous and breast tissue in ultrasound images by incorporating ultrasound RF waveform data. Med. Image Anal..

[56] Maghsoudi O.H., Christopher S., Gastounioti A., Pantalone L., Wu F.-F., Cohen E.A., Stacey W., Conant E.F., Vachon C., Kontos D. (2021). Abstract 2600: Deep-LIBRA: An artificial intelligence approach for fully-automated assessment of breast density in digital mammography. Cancer Res..

[57] Maghsoudi O.H., Gastounioti A., Scott C., Pantalone L., Wu F.-F., Cohen E.A., Winham S., Conant E.F., Vachon C., Kontos D. (2021). Deep-LIBRA: An artificial-intelligence method for robust quantification of breast density with independent validation in breast cancer risk assessment. Med. Image Anal..

[58] He K., Sun J. Convolutional neural networks at constrained time cost. Proceedings of the IEEE conference on computer vision and pattern recognition.

[59] Chen T., Lin L., Zuo W., Luo X., Zhang L. Learning a wavelet-like auto-encoder to accelerate deep neural networks. Proceedings of the AAAI Conference on Artificial Intelligence.

