# Supplementary material for: Dual-Intended Deep Learning Model for Breast Cancer Diagnosis in Ultrasound Imaging
Source: Cancers (Basel). 2022 May 27;14(11):2663. doi: 10.3390/cancers14112663 (PMC9179519; doi:10.3390/cancers14112663)
Supplement: Supplementary file 1 [file cancers-14-02663-s001.zip › cancers-1732153-supplementary.pdf]

cancers MDPI

Supplementary Materials: Dual-Intended Deep Learning Model for Breast Cancer Diagnosis in Ultrasound Imaging

Nicolle Vigil, Madeline Barry, Arya Amini, Moulay Akhloufi, Xavier P.V. Maldague, Lan Ma, Lei Ren, Bardia Yousefi

Table S1. Computational time of the designed deep learning model.

Computational	Time	Memory
**Loading hyperparameters to Deep Convolutional Autoencoder structure**	5.8 secs	2.8 MByte
**One epoch of the model Batch size = 8**	86.5 secs	8.7 MByte
**Extracting Deep-radiomics**	6.3 secs	8.9 MByte
**SpectralEmbedding**	0.1 secs	9.0 MByte
**Random Forest**	0.2 secs	2.6 MBtye

Table S2. More detailed information about the Radiomic features used in this study.

Radiomic features	information
**Quantization method**	Fixed-bin width
**Bin-width size**	25
**Median bin counts (range) for original image**	90 (40–140)
**Median bin counts (range) for all the filters**	85 (30–120)
**Laplcian of Gaussian (LoG)**	LoG with 10 sigma levels (0-5**mm**, strides of 0.5***mm***) For each level, 18 features from first-order statistics were extracted
**Wavelet**	Three channels For each level, 18 features from first-order statistics were extracted

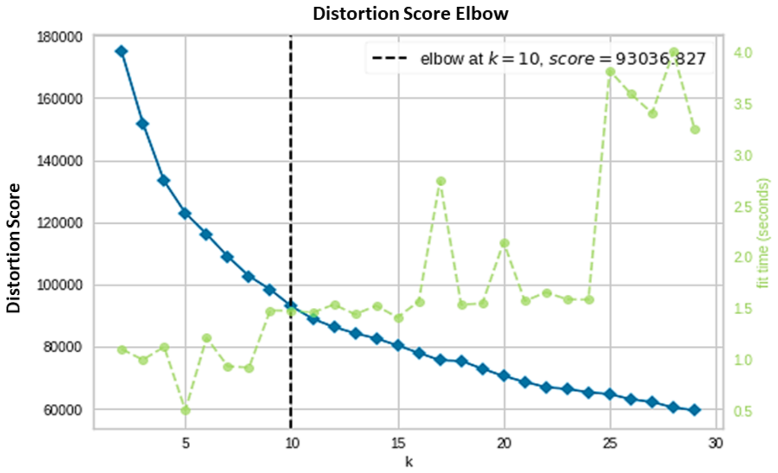

Figure S1. Elbow technique to calculate the distortion score and find out the optimum number of the cluster for conventional radiomics. The k = 10 and k = 12 are both optimum numbers, shown by the graph, where we select k = 12 for this study.

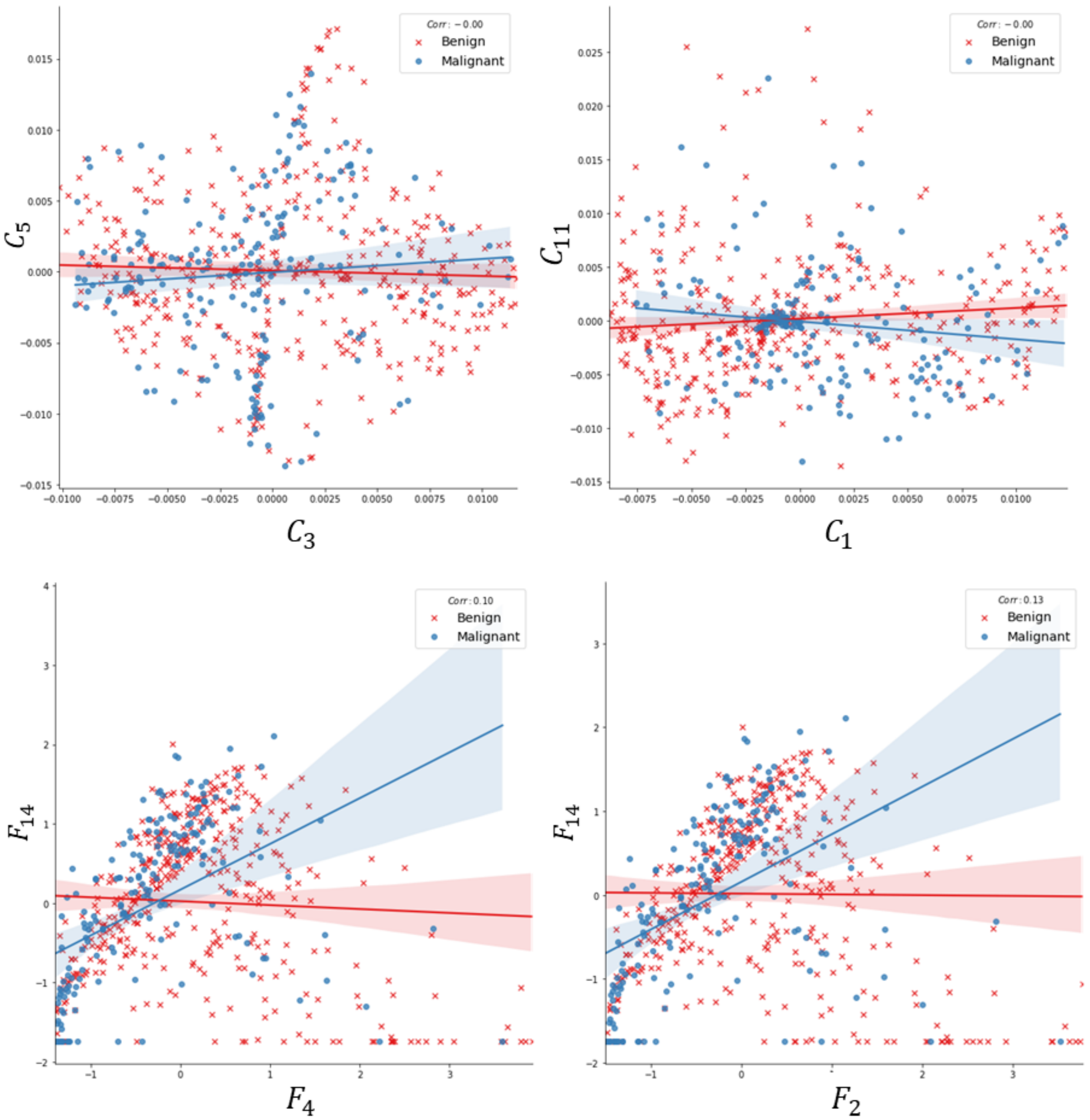

Figure S2. Regression analysis for benign and malignant lesions for four conventional radiomics and four deep radiomics show the significant dissimilarity of the paired feature groups stating independence of these features as predictive imaging biomarkers.

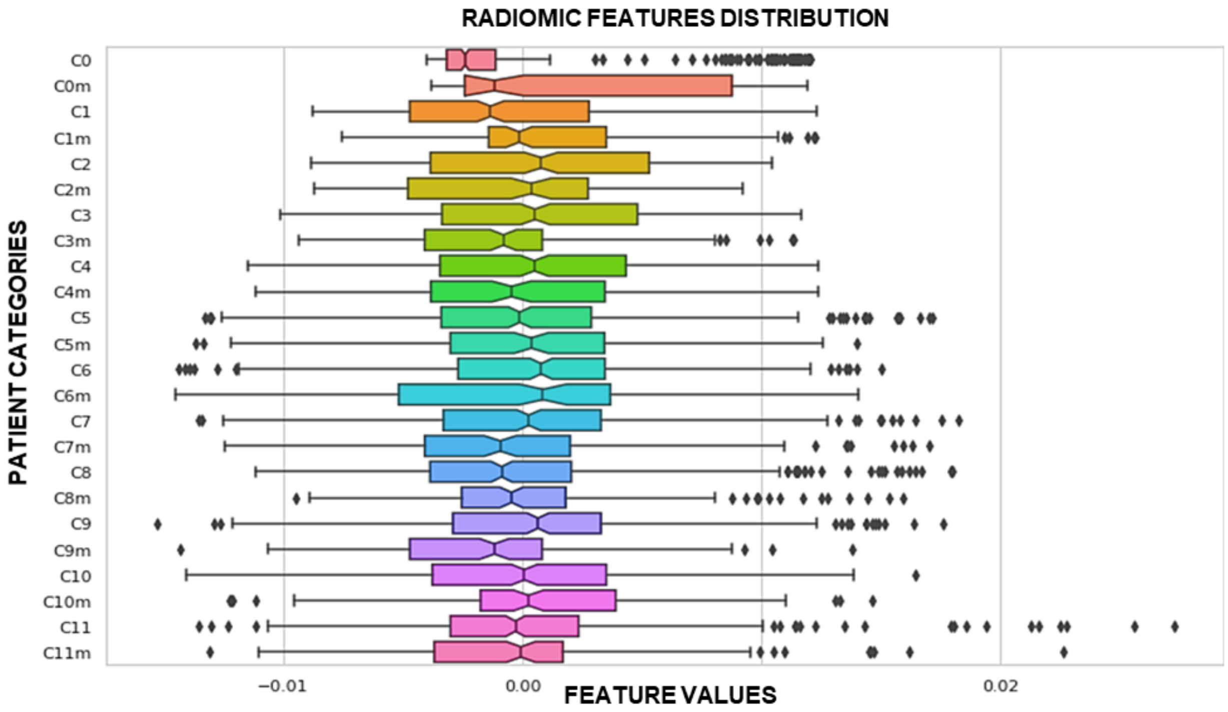

Figure S3. Distribution of conventional radiomics and their effect on diagnosis for malignant and benign lesions are presented in the following boxplots for 12 radiomic groups. Two subsets of patients with malignant lesions, shown by "m" versus benign lesions, without any suffix are compared statistically using the Wilcoxon test.

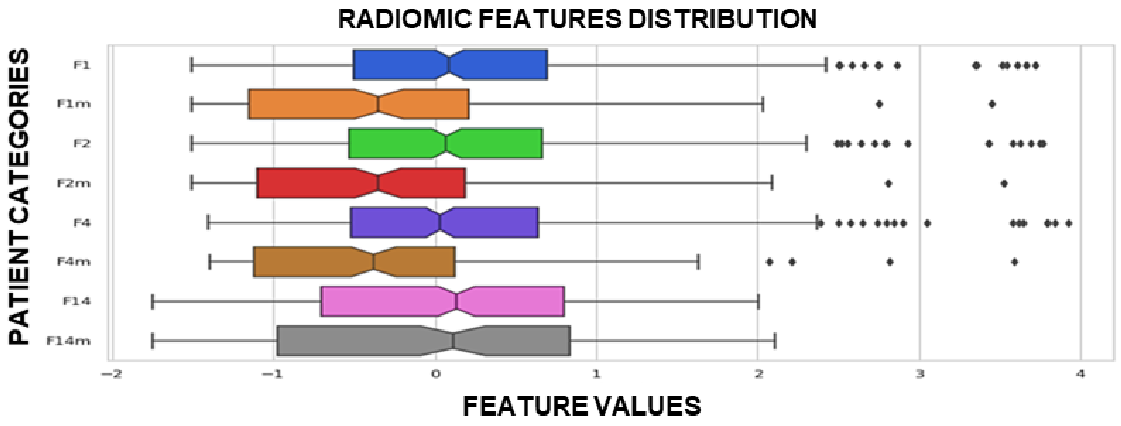

Figure S4. The distribution of Deep radiomics and their classification strength are presented in the following boxplots for two subsets of patients with the malignant lesion, shown by "m" versus benign lesions. This analysis is performed using the Wilcoxon test, which presents the significance statistic among radiomics generated by the proposed deep neural network model.

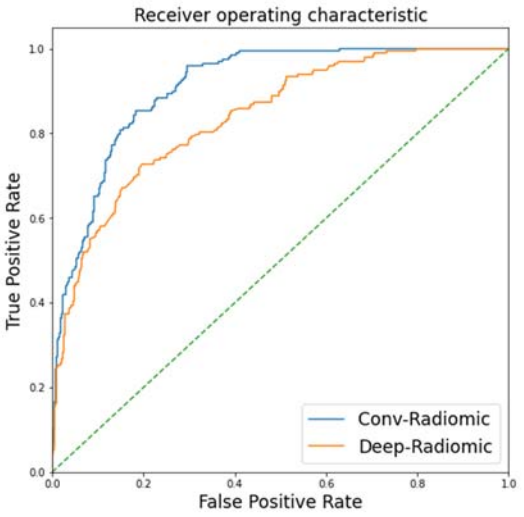

Figure S5. The receiver operating characteristic (ROC) curves for different multivariant models using conventional and deep radiomic features. Vertical and horizontal axes in the ROC represent sensitivity and 1- specificity of the model.

S1. Grid Search Hyperparameter Tunning

We have performed hyperparameter tunning using Grid search algorithm for a range of given hyperparameters applying K-fold cross-validation. For k = 5, our maximal random forest model was challenged by adding different range of number of the trees (10-128), maximum depth (2-7), and random state (40-80). This led to suggested systems' parameters, No. est. = 16, Max depth = 4, Rand. state = 80, yielded an accuracy of 75.3% (62.5%-80.4%), which very close to the reported accuracy for leave-one-out cross-validation for the closely related parameters (See Table 2). Table S3 presents the hyperparameter scores obtained by the model and their scores and optimization time.

Table S3. The hyperparameter scoring obtained by grid search and 5-fold cross validation of the model and their scores and optimization time.

Grid Search Hyperparameter tunning							
**Max Depth**	**No. Est.**	**Rand. State**	**Average Fitting Time**	**STD Fitting Time**	**Mean Test Score**	**STD Test Score**	**Rank Test Score**
2	10	40	0.017	0.002	0.674	0.056	138
2	10	50	0.015	0.001	0.677	0.063	126
2	10	60	0.019	0.002	0.665	0.041	158
2	10	70	0.018	<0.001	0.66	0.05	160
2	10	80	0.017	0.001	0.679	0.037	110
2	16	40	0.026	<0.001	0.682	0.042	98
2	16	50	0.026	<0.001	0.679	0.058	110
2	16	60	0.025	<0.001	0.666	0.039	156
2	16	70	0.027	0.002	0.665	0.049	158
2	16	80	0.026	0.002	0.671	0.054	145
2	20	40	0.035	0.003	0.683	0.045	90
2	20	50	0.033	0.002	0.679	0.061	110
2	20	60	0.03	<0.001	0.677	0.046	128
2	20	70	0.03	0.003	0.669	0.056	149
2	20	80	0.03	0.001	0.674	0.05	138
2	32	40	0.051	0.003	0.687	0.051	71
2	32	50	0.047	0.002	0.674	0.054	138
2	32	60	0.047	0.001	0.677	0.05	128
2	32	70	0.048	0.002	0.668	0.059	153

2	32	80	0.054	0.002	0.68	0.046	106
2	64	40	0.097	0.004	0.683	0.056	90
2	64	50	0.1	0.005	0.668	0.059	153
2	64	60	0.098	0.004	0.674	0.052	135
2	64	70	0.105	0.005	0.669	0.056	149
2	64	80	0.091	0.001	0.679	0.045	120
2	70	40	0.102	0.002	0.682	0.055	98
2	70	50	0.108	0.011	0.671	0.06	146
2	70	60	0.102	0.001	0.672	0.05	141
2	70	70	0.105	0.005	0.669	0.056	149
2	70	80	0.102	0.002	0.679	0.045	120
2	80	40	0.118	0.003	0.679	0.053	120
2	80	50	0.117	0.002	0.668	0.062	153
2	80	60	0.12	0.005	0.672	0.059	141
2	80	70	0.126	0.007	0.671	0.053	146
2	80	80	0.12	0.003	0.679	0.045	120
2	128	40	0.194	0.01	0.68	0.05	106
2	128	50	0.244	0.062	0.666	0.062	156
2	128	60	0.19	0.003	0.672	0.055	141
2	128	70	0.193	0.004	0.671	0.057	146
2	128	80	0.192	0.009	0.676	0.051	132
4	10	40	0.019	0.003	0.674	0.08	135
4	10	50	0.018	<0.001	0.685	0.075	81
4	10	60	0.018	<0.001	0.682	0.066	101
4	10	70	0.018	0.001	0.687	0.078	71
4	10	80	0.02	<0.001	0.691	0.091	32
4	16	40	0.031	0.001	0.674	0.082	135
4	16	50	0.028	0.002	0.679	0.061	110
4	16	60	0.028	0.002	0.672	0.082	141
4	16	70	0.028	<0.001	0.687	0.086	71
4	16	80	0.031	0.002	0.707	0.081	1
4	20	40	0.038	0.001	0.683	0.079	90
4	20	50	0.037	0.001	0.685	0.071	81
4	20	60	0.039	0.004	0.687	0.067	78
4	20	70	0.034	0.002	0.691	0.078	32
4	20	80	0.034	0.001	0.706	0.082	2
4	32	40	0.056	0.002	0.691	0.077	32
4	32	50	0.061	0.004	0.691	0.065	32
4	32	60	0.057	0.001	0.696	0.072	14
4	32	70	0.053	<0.001	0.688	0.088	53
4	32	80	0.059	0.005	0.688	0.083	53

4	64	40	0.107	0.003	0.693	0.079	23
4	64	50	0.109	0.003	0.688	0.074	53
4	64	60	0.118	0.005	0.693	0.08	23
4	64	70	0.111	0.006	0.696	0.089	14
4	64	80	0.11	0.004	0.696	0.083	14
4	70	40	0.132	0.004	0.693	0.079	23
4	70	50	0.125	0.008	0.688	0.071	53
4	70	60	0.121	0.006	0.693	0.074	23
4	70	70	0.123	0.007	0.691	0.086	32
4	70	80	0.124	0.007	0.702	0.078	4
4	80	40	0.142	0.004	0.698	0.075	10
4	80	50	0.146	0.007	0.688	0.078	53
4	80	60	0.137	0.004	0.687	0.082	71
4	80	70	0.143	0.008	0.69	0.086	48
4	80	80	0.147	0.006	0.702	0.08	4
4	128	40	0.224	0.017	0.691	0.074	32
4	128	50	0.217	0.007	0.688	0.087	53
4	128	60	0.226	0.011	0.679	0.084	110
4	128	70	0.222	0.008	0.688	0.085	53
4	128	80	0.225	0.009	0.693	0.081	23
5	10	40	0.028	0.003	0.704	0.078	3
5	10	50	0.044	0.015	0.688	0.068	53
5	10	60	0.029	0.003	0.677	0.064	126
5	10	70	0.02	0.002	0.682	0.077	98
5	10	80	0.018	<0.001	0.688	0.063	53
5	16	40	0.029	0.001	0.699	0.09	6
5	16	50	0.029	<0.001	0.687	0.076	71
5	16	60	0.029	0.001	0.679	0.081	110
5	16	70	0.029	0.002	0.69	0.087	48
5	16	80	0.032	0.001	0.69	0.068	48
5	20	40	0.037	0.002	0.698	0.088	10
5	20	50	0.035	0.001	0.699	0.074	6
5	20	60	0.035	0.001	0.683	0.091	90
5	20	70	0.034	<0.001	0.691	0.084	32
5	20	80	0.04	0.003	0.699	0.083	6
5	32	40	0.063	0.002	0.691	0.087	32
5	32	50	0.062	<0.001	0.691	0.082	32
5	32	60	0.061	0.001	0.687	0.093	71
5	32	70	0.057	0.003	0.688	0.087	53
5	32	80	0.056	0.002	0.696	0.089	14
5	64	40	0.112	0.004	0.68	0.084	106
5	64	50	0.119	0.002	0.687	0.088	78

5	64	60	0.111	0.002	0.679	0.085	110
5	64	70	0.111	0.002	0.699	0.083	6
5	64	80	0.11	0.002	0.685	0.097	81
5	70	40	0.122	0.002	0.693	0.082	23
5	70	50	0.122	0.003	0.685	0.091	88
5	70	60	0.128	0.005	0.679	0.088	110
5	70	70	0.133	0.004	0.696	0.089	14
5	70	80	0.121	0.001	0.682	0.097	101
5	80	40	0.14	0.002	0.688	0.083	53
5	80	50	0.152	0.006	0.691	0.084	32
5	80	60	0.14	0.002	0.679	0.087	110
5	80	70	0.15	0.009	0.693	0.087	23
5	80	80	0.14	0.003	0.691	0.095	32
5	128	40	0.237	0.008	0.683	0.086	90
5	128	50	0.224	0.004	0.685	0.082	81
5	128	60	0.225	0.002	0.679	0.094	110
5	128	70	0.222	0.002	0.696	0.089	14
5	128	80	0.232	0.005	0.688	0.093	53
6	10	40	0.02	0.001	0.688	0.087	53
6	10	50	0.022	0.001	0.685	0.077	81
6	10	60	0.021	0.001	0.669	0.086	149
6	10	70	0.021	0.001	0.676	0.089	132
6	10	80	0.021	0.001	0.68	0.072	106
6	16	40	0.033	<0.001	0.688	0.098	53
6	16	50	0.033	0.001	0.685	0.079	88
6	16	60	0.032	0.001	0.677	0.093	128
6	16	70	0.033	0.001	0.698	0.103	13
6	16	80	0.03	0.001	0.688	0.072	53
6	20	40	0.036	<0.001	0.693	0.095	23
6	20	50	0.037	0.001	0.688	0.079	53
6	20	60	0.036	0.001	0.691	0.098	47
6	20	70	0.04	0.003	0.679	0.098	120
6	20	80	0.04	0.001	0.676	0.084	132
6	32	40	0.065	0.003	0.698	0.105	10
6	32	50	0.065	0.003	0.694	0.081	22
6	32	60	0.059	0.001	0.682	0.099	101
6	32	70	0.058	0.001	0.683	0.101	95
6	32	80	0.059	0.002	0.687	0.081	71
6	64	40	0.118	0.005	0.691	0.108	32
6	64	50	0.122	0.004	0.682	0.091	101
6	64	60	0.117	0.003	0.696	0.105	14
6	64	70	0.115	0.002	0.683	0.093	95
6	64	80	0.117	0.002	0.679	0.101	120
6	70	40	0.129	0.002	0.696	0.097	14

6	70	50	0.129	0.003	0.682	0.092	101
6	70	60	0.135	0.004	0.685	0.096	81
6	70	70	0.13	0.003	0.688	0.09	53
6	70	80	0.127	0.003	0.677	0.093	128
6	80	40	0.155	0.007	0.693	0.1	23
6	80	50	0.149	0.008	0.683	0.092	95
6	80	60	0.149	0.005	0.691	0.1	32
6	80	70	0.149	0.005	0.688	0.094	53
6	80	80	0.146	0.005	0.691	0.099	32
6	128	40	0.241	0.01	0.69	0.092	52
6	128	50	0.242	0.012	0.687	0.094	78
6	128	60	0.234	0.004	0.69	0.104	48
6	128	70	0.247	0.011	0.691	0.09	32
6	128	80	0.235	0.007	0.685	0.1	81
